# The Effect of Oxygen Partial Pressure on Microstructure and Properties of Fe40Al Alloy Sintered under Vacuum

**DOI:** 10.3390/ma8041513

**Published:** 2015-03-31

**Authors:** Dariusz Siemiaszko, Beata Kowalska, Paweł Jóźwik, Monika Kwiatkowska

**Affiliations:** Department of Advanced Materials and Technologies, Faculty of Advanced Technologies and Chemistry, Military University of Technology, Kaliskiego 2, 00-908 Warsaw, Poland; E-Mails: beatak87@gmail.com (B.K.); pjozwik@wat.edu.pl (P.J.); mkwiatkowska@wat.edu.pl (M.K.)

**Keywords:** iron aluminides, phase identification, oxides, vacuum, reaction synthesis

## Abstract

This paper presents the results of studies on the influence of oxygen partial pressure (vacuum level in the chamber) on the properties of FeAl intermetallics. One of the problems in the application of classical methods of prepared Fe-Al intermetallic is the occurrence of oxides. Applying a vacuum during sintering should reduce this effect. In order to analyze the effect of oxygen partial pressure on sample properties, five samples were processed (by a pressure-assisted induction sintering—PAIS method) under the following pressures: 3, 8, 30, 80, and 300 mbar (corresponding to oxygen partial pressures of 0.63, 1.68, 6.3, 16.8, and 63 mbar, respectively). The chemical and phase composition, hardness, density, and microstructure observations indicate that applying a vacuum significantly impacts intermetallic samples. The compact sintered at pressure 3 mbar is characterized by the most homogeneous microstructure, the highest density, high hardness, and nearly homogeneous chemical composition.

## 1. Introduction

Alloys based on the Fe-Al intermetallic phases can be prepared by conventional methods, such as melting, casting, or powder metallurgy; however, the preparation of such materials by conventional methods leads to technological problems due to the large difference between the melting points of iron (1535 °C) and aluminum (660 °C), cross-reactivity, and strong affinity for oxygen [1,2,3]. One of the major problems in the application of classical methods is that the starting powders (Fe and Al) must be dried before melting, otherwise aluminum combines with water vapor, resulting in the production of large quantities of hydrogen, which is then dissolved in the liquid metal and is the cause of the formation of gas pores during casting [4,5].

Another problem in applying classical methods is the occurrence of oxides. Powders of iron and aluminum that are stored in air are covered with a layer of oxide (resulting in a negative free energy of formation of both iron oxide and aluminum oxide). During the formation of FeAl, iron oxides are reduced according to the scheme [6]:

(1) $$3\text{FeO}+2\text{Al}\to 3\text{Fe}+{\text{Al}}_{2}{\text{O}}_{3},\;\;\text{ΔH}=\;-880\text{ kJ}$$

(2) $${\text{Fe}}_{2}{\text{O}}_{3}+2\text{Al}\to 2\text{Fe}+{\text{ Al}}_{2}{\text{O}}_{3},\text{ ΔH}=\;-850\text{ kJ}$$

(3) $$3{\text{Fe}}_{3}{\text{O}}_{4}+8\text{Al}\to 9\text{Fe}+\;4{\text{Al}}_{2}{\text{O}}_{3},\text{ ΔH}=\;-3350\text{ kJ}$$

Increasing the proportion of the Al_2_O_3_ precipitates in the finished sample. Simultaneously, on the surface of aluminum powder, there are reduction-resistant, continuous Al_2_O_3_ films. Their presence is an important barrier that hinders the interdiffusion of iron and aluminum, which significantly delays or prevents the complete reconstruction of the structure of orderly-sintered FeAl. To overcome this, Durejko and Bojar [7,8] used a cyclically uniaxial variable load (60 Hz) during sintering. The use of cyclical load at elevated temperature enhances the fragmentation of the oxide films and facilitates diffusion between the grains of iron and aluminum. The sample’s structure after the heat treatment consists of fine oxides of Al_2_O_3_, located at the border of fine grains. Oxidation of the surface is a negative phenomenon due to the deterioration of the mechanical properties of the produced samples [8,9]. The solution to these problems is sintering under vacuum [10,11].

Sintering in a vacuum facilitates removal of the adsorbed gases from the surface of the powder; a sufficiently high vapor pressure reduces the concentration of oxygen, and the thermodynamic conditions protect against oxidation and enable the thermal dissociation of some oxides, nitrides, and hydrides. Applying a vacuum during sintering has also important technological advantages: Vacuum, as an excellent thermal insulator, considerably reduces the heat necessary to heat the chamber, so the process is efficient and saves energy.

A disadvantage of carrying out the sintering process under vacuum is the considerably higher cost of equipment, difficulties in process automation, and the long time needed to pump out a chamber, which increases with increasing vacuum level. The question is: What is the minimum vacuum level necessary to provide adequate purity of sintered FeAl? In addition, the influence of the level of vacuum to changes in the phase composition is unknown.

## 2. Results and Discussion

### 2.1. Characterization of the Iron and Aluminum Powders

**Figure 1:**
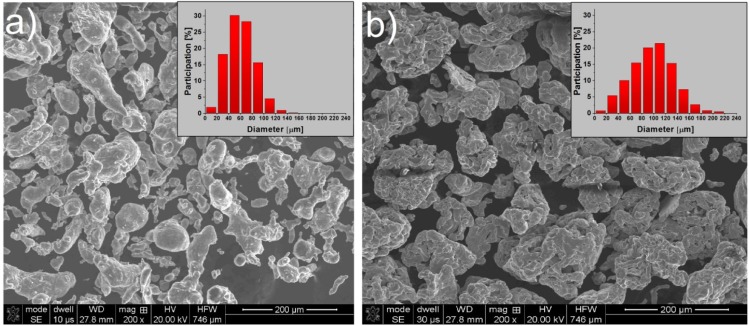
The morphology and particle size distribution of (**a**) aluminum and (**b**) iron powders.

The morphology and particle size distribution of the iron and aluminum powders is shown on Figure 1. The average particle size of the aluminum was *ca.* 60 μm. The iron powder particles were bigger and their average particle size was *ca.* 100 μm.

**Figure 2:**
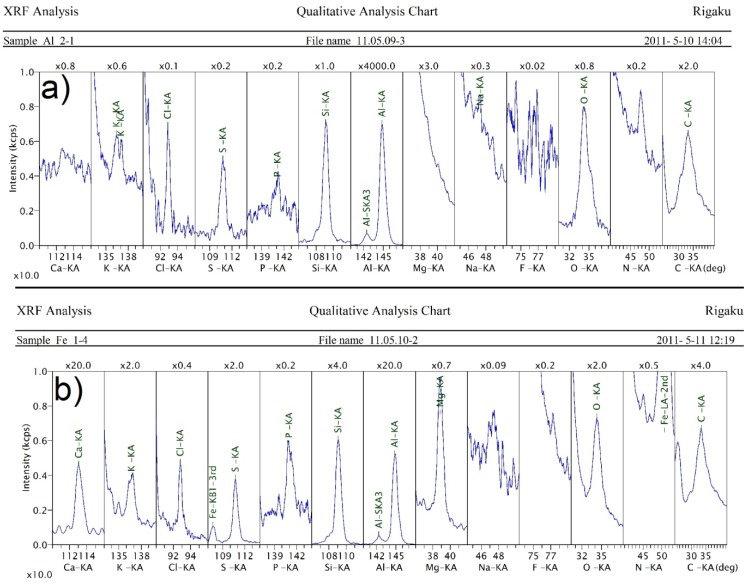
The contaminants of (**a**) aluminum and (**b**) iron powders, measured by X-ray diffraction (XRD).

Table 1 shows the average oxygen content of the starting powders. X-ray fluorescence (XRF) analysis showed that 2.1% of the starting Fe powder’s mass was oxygen, while 2.3% of the starting Al powder’s mass was oxygen. XRF analysis also helped to determine the contaminants present in the powders of Fe and Al (Figure 2). Contamination represents less than 1%, by weight, of the powder. The Al powder had K, Cl, S, P, Si, Na, F and C contamination. For the Fe powder, the impurities were similar except Ca and Mg were also detected. These contaminations were likely introduced to the powder from bad storage (contact with the skin), because they should have been removed during the manufacturing process.

**Table 1:** The average oxygen content of the starting powders.

Method	XRF		EDS	
Power	Mean	Stand. dev.	Mean	Stand. dev.
Fe	2.1%	0.1	1.4%	0.2
Al	2.3%	0.1	1.2%	0.3

### 2.2. Density, Porosity and Hardness of the Samples

**Figure 3:**
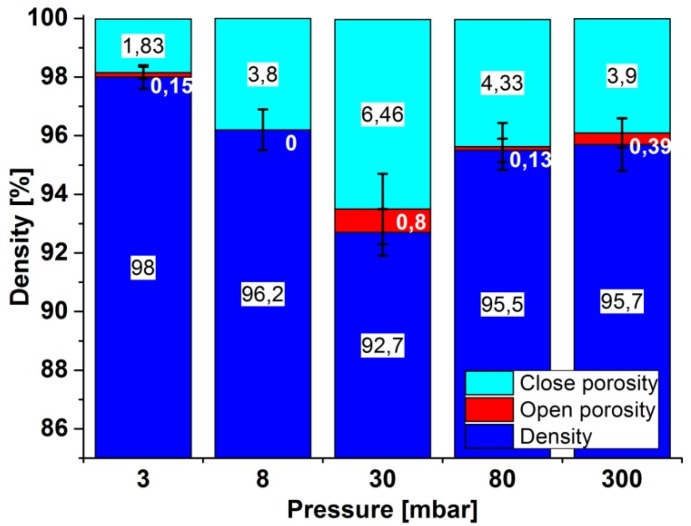
The density and the open and closed porosity of samples.

Figure 3 shows the results of the density measurements and the open and closed porosity of samples after sintering. A slight increase in density with decreasing pressure in the chamber was observed. The largest relative density value (98%) was from a sample sintered at a pressure of 3 mbar, and the lowest density value (92.7%) characterized the specimen generated at a pressure of 30 mbar. However, in this case the standard deviation is highest. A further increase in chamber pressure caused a slight increase in the density of samples, to approximately 95.5%. In almost all the samples, there was no open porosity. Participation of the open porosity in any samples did not exceed 0.4%; however, the absence of open porosity in sintered specimens is no surprise, due to the application of pressure during the self-propagation high-temperature synthesis (SHS) between iron and aluminum. This reaction raises the temperature of the samples temporarily and thus enables deformation of the surface by the action of pressure, closing most open pores.

**Figure 4:**
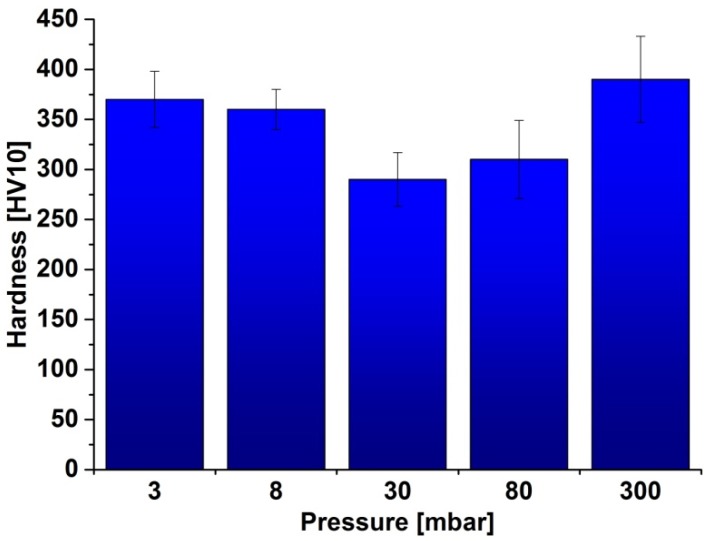
The hardness of the samples.

The hardness of the samples (Figure 4) follows a similar trend. The greatest hardness values were obtained for the samples formed at the extreme values vacuum (3 and 300 mbar). The hardness of these samples reached approx. 370HV10. The lowest hardness characterized the sample sintered at 30 mbar pressure—290HV10.

### 2.3. X-ray Diffraction Analysis

**Figure 5:**
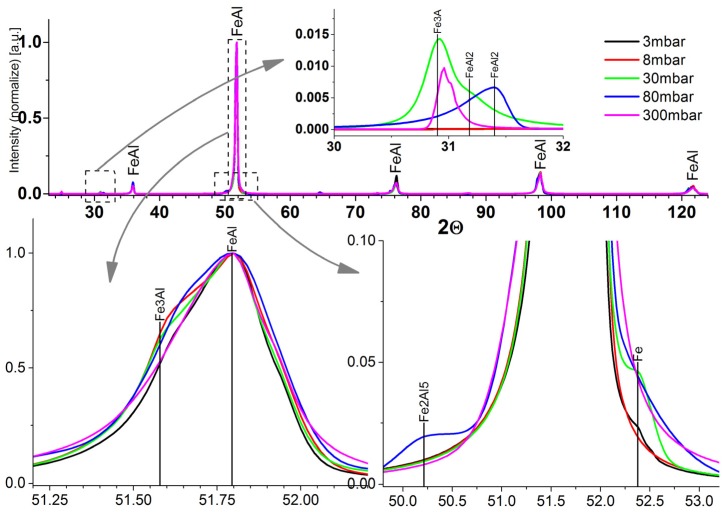
XRD analysis of the samples.

X-ray diffraction (XRD) experiments (Figure 5) showed that all the samples were of similar composition regarding the dominant FeAl phase; there was also a significant share of Fe_3_Al phase. As the pressure in the chamber was reduced, the samples homogenized. The peak Fe_3_Al phase—Angle 30.9° disappeared, as did the phase FeAl_2_—Angles 31.2 and 31.4, and the phase of Fe_2_Al_5_ (50.2°). However, most important is the reduction of the intensity of the iron peak (52.4°). If the pressure in the chamber was greater than 30 mbar, there was a significantly higher share of iron in the sample, suggesting that higher pressure inhibits the reaction between iron and aluminum, which results in a more non-equilibrium structure of the resulting sample. However, the mechanism of this phenomenon still requires further analysis. The compacts sintered at 3 and 8 mbar showed only minor proportions of iron.

### 2.4. Microstructure, Chemical Composition, and Oxide Distribution

**Figure 6:**
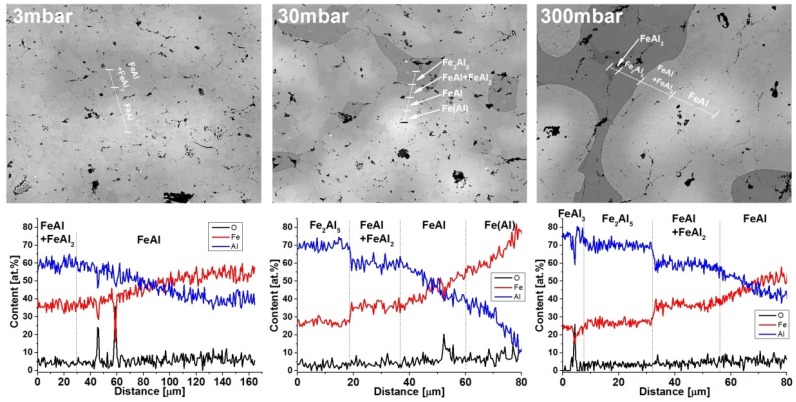
The chemical composition of the samples obtained at pressures of 3, 30, and 300 mbar.

**Figure 7:**
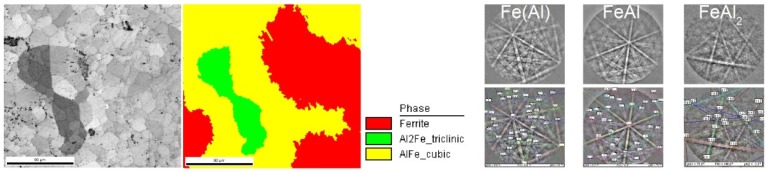
An electron backscatter diffraction (EBSD) pattern of sample obtained at a pressure of 3 mbar.

Figure 6 shows the chemical composition of the selected samples. The presence of the FeAl phase and mixtures of FeAl_2_ were confirmed in all the samples. Diffraction studies showed the presence of only the FeAl_2_ phase in the sample obtained at a pressure of 80 mbar. To confirm the presence of this phase, additional tests of phase composition into a micro-area, using electron backscatter diffraction (EBSD), were performed. EBSD studies showed its presence in all the samples. Figure 7 shows an EBSD image of sample obtained with a pressure of 3 mbar. In the majority of specimens, participation of the FeAl_2_ phase is too small to detect by XRD.

All obtained samples were re-studied to determine the oxygen content (EDS and XRF method) and the presence of trace impurities (XRF). Table 2 summarizes the results of the oxygen content testing. Results obtained by both XRF and EDS showed that the oxygen content of the sample was similar for all the applied pressures. By the XRF method, the oxygen content was approx. 2%, while EDS showed that it was approx. 1.2%.

**Table 2:** The average oxygen content of the samples.

Pressure	3 mbar	8 mbar	30 mbar	80 mbar	300 mbar
XRF	2.0% ± 0.4%	2.3% ± 0.2%	2.6% ± 0.2%	1.7% ± 0.2%	1.6% ± 0.2%
EDS	1.4% ± 0.1%	1.2% ± 0.05%	1.3% ± 0.05%	1.3% ± 0.05%	1.2% ± 0.05%

**Figure 8:**
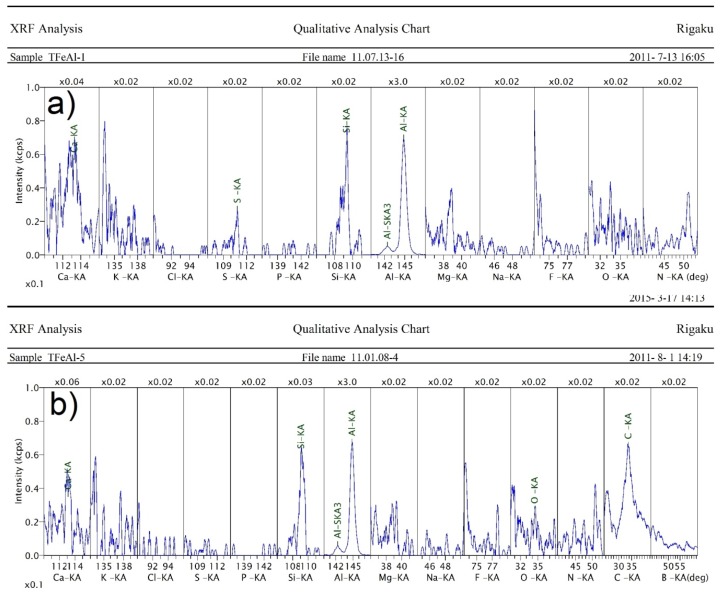
The contaminants of sample obtained at a pressure of (**a**) 3 and (**b**) 300 mbar, measured by XRF.

Re-examinations of the trace contaminants were performed. Figure 8 shows selected (3 and 300 mbar) XRF analyses. Regardless of the pressure of sintering, there was no presence of impurities (except Si and C under 300 mbar); this result confirms the beneficial effect of the vacuum on the samples’ purity. Reducing the pressure during the sintering reaction increases the vapor pressure of elements and thus their removal from the finished product.

**Figure 9:**
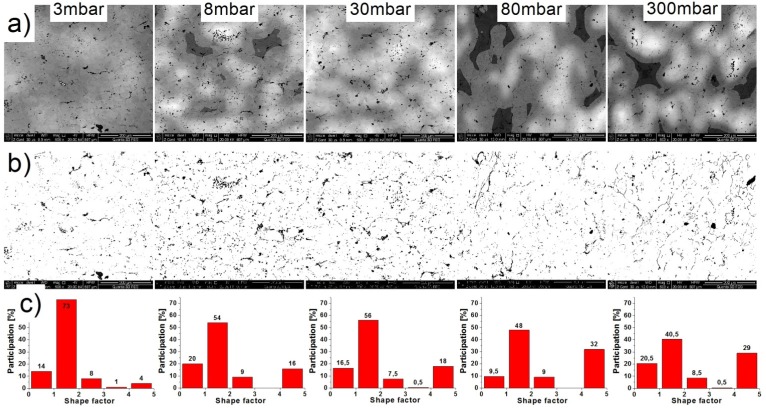
(**a**) The microstructure images; (**b**) image after binarization; (**c**) and the shape factor distributions of the porosity/oxides of the samples.

Microstructure observation (Figure 9a) confirmed the importance of the chamber pressure on the homogeneity of the sample. As shown in the pictures, decreasing pressure decreased the participation of the bright areas (with high concentrations of iron), *i.e.*, increasing the homogeneity of the samples. The reasons for this influence of the pressure level on the homogeneity of samples are not clear. Below, there is proposed mechanism to explain this phenomenon, but further research is required to confirm it empirically. It is well-known that plasticization of aluminum takes place during the SHS reaction. Simultaneously, pressured vapor is locked in the pores and tries to get out towards the vacuum. The motion of the air molecules may force a more uniform distribution of the aluminum and thereby provide a better homogeneity of the sample after the reaction.

**Figure 10:**
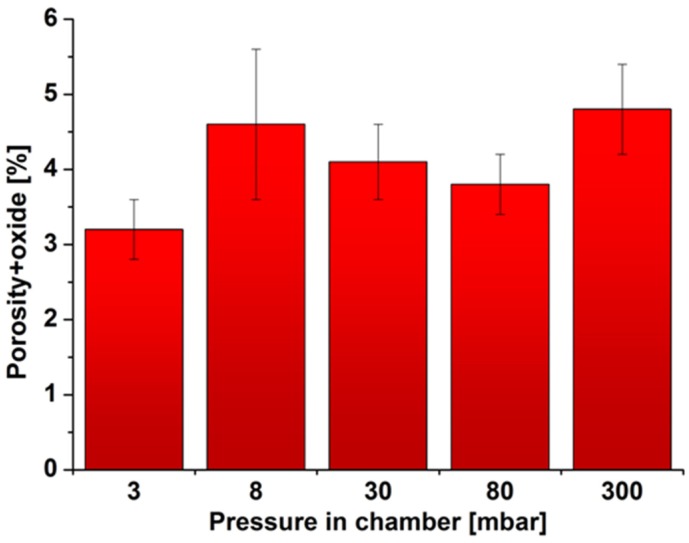
The amount of porosity/oxides in the samples, depending on the vacuum levels.

Figure 9b shows an exemplary image of the microstructure after binarization. Black areas represent the pores and oxides. Because of the similar shape and shade, it was not possible to separate these two elements on the output images. Analyzing the resulting images, the amount of porosity/oxides is the lowest for 3 mbar pressure but is similar in other cases. These observations were confirmed by quantitative image analysis (Figure 10). The share of porosity and oxides in the first sample (3 mbar) is approx. 3%, while others range from 4% to 4.5%. The differences in the results for the porosity, measured by the Archimedes method and image analysis, were due to the different methods of measurement. Archimedes' method determines the average porosity of the entire sample, whereas in the second method, several images are analyzed (in this case, eight) at one cross-section of the sample. In addition, we cannot determine what share of the results had the same porosity and what oxides.

**Figure 11:**
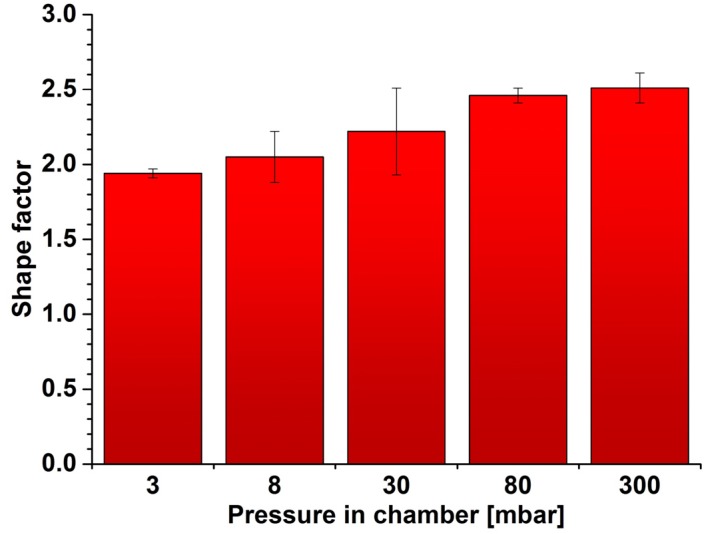
The change of the average shape factor, depending on the vacuum levels.

During analysis, the binary images showed that with increasing pressure, the dark areas are linked together to form long chains. This confirms the average aspect ratio shown in Figure 11. This factor increases from 1.9 (3 mbar) to 2.4 (300 mbar). The increase seems to be small but the predominant share in all the images are small particles, whose shape factor is low; the change of the average shape factor must therefore be regarded as significant. Changes of the average shape factor are more clearly seen by comparing the shape factor distributions (Figure 9c). The share of the elements with a shape factor of 1 to 2 decreased approx. 50% (from 73% to 40%) while the share of the elements in the range of 4–5 increased eight times (from 4% to 30%). The observed effect is not a favorable phenomenon; this causes weakening of grain boundaries and lowers the mechanical properties of the finished product.

**Figure 12:**
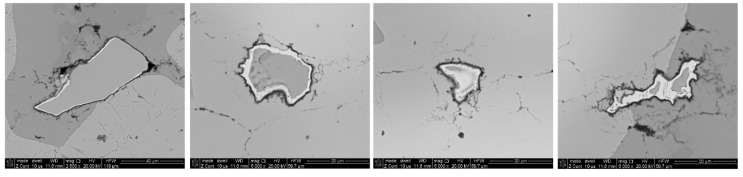
Typical images of the oxide particles with a bright-appearing border inside the samples.

**Figure 13:**
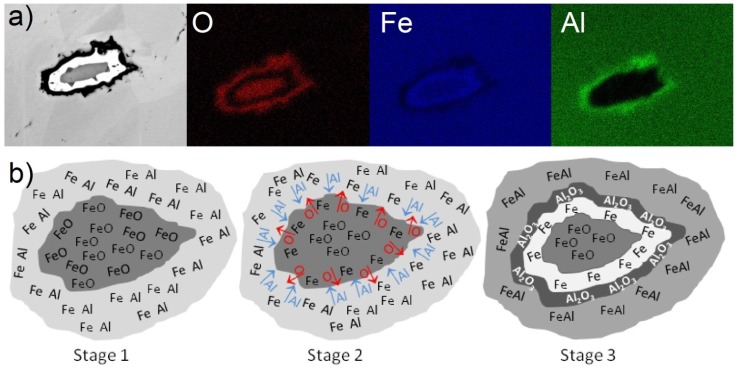
(**a**) EDS analysis of typical oxide particle and (**b**) the proposed mechanism of formation of this type of particle.

During the analysis of the microstructure in all samples, oxide particles with a characteristic bright border were observed. Typical examples of the oxide particles are shown in Figure 12. As shown, around all of the particles we can see a bright-appearing border; EDS analysis (Figure 13a) showed that it is a layer of iron. Simultaneously, outside the iron layer, a thin layer of oxygen-rich aluminum was detected.

Figure 13b presents the proposed mechanism for the formation of the bright border of iron and the layer of oxygen-rich aluminum. Initially, the iron oxide particle is surrounded by a mixture of iron and aluminum (Stage 1). When heated to temperatures close to the melting point of aluminum, the SHS reaction with iron starts. The heat released during the SHS reaction is sufficient to initiate local thermite reaction (according to equation 1). Aluminum reacts with the iron oxide on the surface of the particle, binding oxygen (Stage 2). Because the reaction of the FeAl formation exhausted “resources” like pure aluminum, the thermite reaction is stopped. This process results in the formation of layers made of iron and aluminum oxide between the iron oxide particles and FeAl area (Stage 3).

## 3. Materials and Methods

Starting materials were commercial powders of iron and aluminum, stored in the air. Iron powder was under the trade name NC 100.24 (Höganäs, Höganäs, Sweden) and aluminum powder was under the trade name AG 90/99.7 (Benda-Lutz, Skawina, Poland). The data regarding the powders are summarized in Table 3. Iron and aluminum powders, in an atomic ratio of Fe:Al—60:40, were mixed in a Turbula mixer for 30 min, and then compacted under 50 MPa load. Green compact, with a diameter of 50 mm and a height of approx. 10 mm, was placed in a graphite die and a sintering process was performed. During compaction, air could remain in the closed pores in the samples. Oxygen contained therein could react with iron, leading to further oxidation; however, because all samples were compacted under identical conditions, the effect was comparable for all samples. Thus, during the analysis, this effect could be omitted. In order to analyze the effect of the vacuum level on the sample’s properties, five processes, under following pressures, 3, 8, 30, 80, and 300 mbar, were performed; these pressures corresponded to the following partial pressures of oxygen: 0.63, 1.68, 6.3, 16.8, and 63 mbar. Other process parameters are summarized in Table 4.

**Table 3:** The data of the iron and aluminum powders.

Powder	The trade name	Average particle size	Percentile 10%	Percentile 90%	BET surface
		[μm]	[μm]	[μm]	[m^2^/g]
Aluminum	AG 90/99.7	60.1	31.5	92.9	0.99
Iron	NC 100.24	101.2	48.9	143.5	0.07

**Table 4:** Process parameters during PAIS sintering.

Sintering temperature [°C]	Load [MPa]	Annealing time [min]	Average heating rate [°C/min]
1000	60	5	400

**Figure 14:**
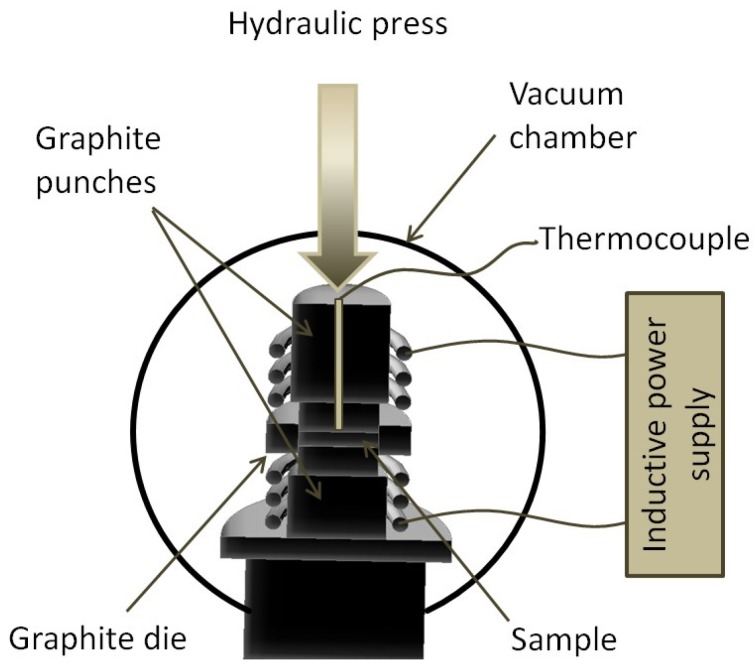
Pressure-assisted induction sintering (PAIS) device scheme.

Sintering is carried out by a PAIS method. Figure 14 shows the schematic of the PAIS device. More information can be found in [12]. During sintering, temperature was measured by thermocouple in the axis of the sample, 1 mm from the surface, as shown in [13].

Density and porosity of samples were tested by the Archimedes method in water (theoretical density of Fe40Al is 6.06 g/cm^3^ [14]). A Vickers hardness test with a load of 98 N was performed. The microstructure was observed by a Scanning Electron Microscope 3D Quanta FEG (FEI, Hillsboro, OR, USA) with BSE detector. Selected images were subjected to binarization and the share of oxides and porosity in the compacts were calculated. For each sample, eight images in different areas of the sample were analyzed and the results were averaged.

The chemical composition was investigated by two methods. To determine the content of the oxides and other impurities in the powders and samples, XRF was used via a Rigaku Primus II and by EDS, using a Scanning Electron Microscope Quanta 3D FEG.

Research on phase composition was carried out using XRD, using an X-ray diffractometer Ultima IV (Rigaku, Osaka, Japan) using a cobalt lamp. Diffraction patterns were collected using Co radiation in parallel beam optics mode. Continuous scanning, 1 deg/min, was performed with sampling of each 0.02 deg. Linear detector was used (Detex ultra, Rigaku, Osaka, Japan). Before starting the analysis, all the obtained diffraction patterns were normalized. Normalization consisted of dividing all obtained measurement points by the highest intensity in the pattern. Studies of phase composition in a micro-area were performed using the EBSD method.

## 4. Conclusions

From the analysis described above, the following conclusions can be drawn: Applying a vacuum has a significant impact on the microstructure and properties of the obtained sintered Fe40Al; sintering at a pressure of 3000 mbar reduces the oxygen content in the final material; and applying a vacuum enables the removal of trace impurities. Simultaneously, however, undesirable elements in the form of oxides and porosity formed elongated shaped regions that may reduce the strength properties; only carrying out sintering at lower pressures limits that unfavorable phenomenon.

Pressure reduction also causes substantial improvements of the microstructure and phase composition. Reducing pressure below 30 mbar eliminates unreacted iron and the unfavorable phases with high aluminum content (FeAl_3_ and Fe_2_Al_5_). It also limits the participation of the Fe_3_Al phase. The optimal pressure to consolidation was 3 mbar. The sample obtained at that pressure was characterized by the most homogeneous microstructure, the highest density, high hardness, and nearly homogeneous chemical composition. In order to remove insignificant quantities of Fe_2_Al phase and uniform distribution of iron in the phase Fe40Al, further annealing is necessary. Because iron is no longer present in samples obtained that way, the homogenizing process may be conducted in air. In addition, it has been shown that the SHS reaction between iron and aluminum may result in a reduction of primary iron oxides occurring in a green compact.
